# *In Vivo* Imaging of Hypoxia and Neoangiogenesis in Experimental Syngeneic Hepatocellular Carcinoma Tumor Model Using Positron Emission Tomography

**DOI:** 10.1155/2020/4952372

**Published:** 2020-08-07

**Authors:** Adrienn Kis, Judit P. Szabó, Noémi Dénes, Adrienn Vágner, Gábor Nagy, Ildikó Garai, Anikó Fekete, Dezső Szikra, István Hajdu, Orsolya Matolay, Gábor Méhes, Gábor Mező, István Kertész, György Trencsényi

**Affiliations:** ^1^Division of Nuclear Medicine and Translational Imaging, Department of Medical Imaging, Faculty of Medicine, University of Debrecen, Nagyerdei St. 98, H-4032 Debrecen, Hungary; ^2^Doctoral School of Clinical Medicine, Faculty of Medicine, University of Debrecen, Nagyerdei St. 98, H-4032 Debrecen, Hungary; ^3^Gyula Petrányi Doctoral School of Allergy and Clinical Immunology, Faculty of Medicine, University of Debrecen, Nagyerdei St. 98, H-4032 Debrecen, Hungary; ^4^Scanomed Ltd, Nagyerdei St. 98, H-4032 Debrecen, Hungary; ^5^Doctoral School of Pharmaceutical Sciences, Faculty of Medicine, University of Debrecen, Nagyerdei St. 98, H-4032 Debrecen, Hungary; ^6^Department of Pathology, Faculty of Medicine, University of Debrecen, Nagyerdei St. 98, H-4032 Debrecen, Hungary; ^7^MTA-ELTE, Research Group of Peptide Chemistry, Hungarian Academy of Sciences, Eötvös L. University, Budapest, Hungary

## Abstract

**Introduction:**

Hypoxia-induced *α*_*ν*_*β*_3_ integrin and aminopeptidase N (APN/CD13) receptor expression play an important role in tumor neoangiogenesis. APN/CD13-specific ^68^Ga-NOTA-c(NGR), *α*_*ν*_*β*_3_ integrin-specific ^68^Ga-NODAGA-[c(RGD)]_2_, and hypoxia-specific ^68^Ga-DOTA-nitroimidazole enable the *in vivo* detection of the neoangiogenic process and the hypoxic regions in the tumor mass using positron emission tomography (PET) imaging. The aim of this study was to evaluate whether ^68^Ga-NOTA-c(NGR) and ^68^Ga-DOTA-nitroimidazole allow the *in vivo* noninvasive detection of the temporal changes of APN/CD13 expression and hypoxia in experimental He/De tumors using positron emission tomography.

**Materials and Methods:**

5 × 10^6^ hepatocellular carcinoma (He/De) cells were used for the induction of a subcutaneous tumor model in Fischer-344 rats. He/De tumor-bearing animals were anaesthetized, and 90 min after intravenous injection of 10.2 ± 1.1 MBq ^68^Ga-NOTA-c(NGR) or ^68^Ga-NODAGA-[c(RGD)]_2_ (as angiogenesis tracers) or ^68^Ga-DOTA-nitroimidazole (for hypoxia imaging), whole-body PET/MRI scans were performed.

**Results:**

Hypoxic regions and angiogenic markers (*α*_v_*β*_3_ integrin and APN/CD13) were determined using ^68^Ga-NOTA-c(NGR), ^68^Ga-DOTA-nitroimidazole, and ^68^Ga-NODAGA-[c(RGD)]_2_ in subcutaneously growing He/De tumors in rats. ^68^Ga-NOTA-c(NGR) showed the strong APN/CD13 positivity of He/De tumors *in vivo*, by which observation was confirmed by western blot analysis. By the qualitative analysis of PET images, heterogenous accumulation was found inside He/De tumors using all radiotracers. Significantly (*p* ≤ 0.01) higher SUVmean and SUVmax values were found in the radiotracer avid regions of the tumors than those of the nonavid areas using hypoxia and angiogenesis-specific radiopharmaceuticals. Furthermore, a strong correlation was found between the presence of angiogenic markers, the appearance of hypoxic regions, and the tumor volume using noninvasive *in vivo* PET imaging.

**Conclusion:**

^68^Ga-DOTA-nitroimidazole and ^68^Ga-NOTA-c(NGR) are suitable diagnostic radiotracers for the detection of the temporal changes of hypoxic areas and neoangiogenic molecule (CD13) expression, which vary during tumor growth in a hepatocellular carcinoma model.

## 1. Introduction

Nowadays, in clinical and experimental oncology, the tumor angiogenesis and hypoxia are one of the most intensively researched areas. At present, little is known about the temporal variation in the expression of angiogenic markers in tumors, and even less in the context of hypoxia. The ability to visualize the formation of new vessels and hypoxic areas in solid tumors using *in vivo* molecular imaging methods allows the noninvasive monitoring of antiangiogenic treatments and the planning of radiotherapy which are critical in patient survival [1]. In malignant tumors, the reduced blood oxygen tension, the inapt capillary system, or the distance between blood vessels and tumor cells can cause hypoxia [2]. In hypoxic cells, the activated HIF transcriptional factors (HIF-1, HIF-2) can cause increased resistance to apoptosis or to radio- or chemotherapy [3–5]. Furthermore, HIF transcriptional factors also promote the development of metastases and neoangiogenic processes in tumors by activating different genes [6]. In most cases, tumor growth and metastatic capacity depend on angiogenesis [7–9]. Tumor neoangiogenesis is the formation of new blood vessels from a preexistent capillary system. Integrins and aminopeptidase N (CD13) are two of the key molecules of targeting neoangiogenesis in the tumors, and the presence and expression rate of these molecules correlate with the intensity of angiogenesis. Among numerous integrins, the *α*_v_*β*_3_ integrins are highly overexpressed on the surface of several cells that contribute to angiogenesis and tumor progression [10, 11]. *α*_v_*β*_3_ integrins are transmembrane receptors on the surface of endothelial cells and can be targeted with RGD (arginine-glycine-asparagine) motif-containing proteins [12, 13]. Aminopeptidase N (CD13) is a zinc-dependent transmembrane exopeptidase [14]. It can be found in high expression on several tumor cells, for example, melanoma and prostate, ovarian, renal, colon, and pancreas cancers, furthermore on the endothelial cell surface [15–17]. It plays an important role in angiogenesis and enzyme-catalysed degradation of the extracellular matrix, which facilitates the tumor cell invasion through the blood stream, hereby causing metastasis formation [18, 19].

*In vivo* imaging of tumor hypoxia and angiogenesis with positron emission tomography (PET) is playing an increasingly important role in the diagnosis of tumors. In addition, more effective antitumor treatments can be planned by using new, specific radiopharmaceuticals that detect angiogenesis and hypoxia in malignant tumors. Radiopharmacons that are labelled with positron emitting radionuclides (^11^C, ^18^F, and ^68^Ga) are used in PET imaging wherewith the uptake and the biodistribution of the labelled molecule can be detected and quantificated *in vivo* [20]. The most commonly used PET radiopharmaceuticals are ^18^F-FDG, ^11^C-methionine, and ^18^F-FLT which give information about cell metabolism, but they are not specific for hypoxia or proteins and receptors that are overexpressed in tumor-associated neoangiogenesis.

For hypoxia imaging, radiolabelled (*e.g.*, ^18^F and ^68^Ga) nitroimidazoles and its derivatives are widely used in PET imaging. Nitroimidazoles go through bioreduction in cells; furthermore, this process causes anion radicals. In the case of normal oxygen supply, these anion radicals are oxidized and it generates diffusible products. But in lower oxygen supply, this procedure does not take place and the free radicals are bound irreversibly to the intracellular macromolecules [21, 22]. Radiolabelled nitroimidazoles (*e.g.*, ^68^Ga-DOTA-nitroimidazole) accumulate in hypoxic cells, thereby enabling the detection of the hypoxic regions in tumor mass [23].

For PET imaging of tumor-associated neoangiogenesis, radiolabelled NGR and RGD peptide-based radiopharmacons are used widespread. NGR motif (asparagine-glycyl-arginine) is a specific ligand of APN/CD13 receptors [24], while RGD's (arginine-glycine-asparagine) double-ring conformation is a selective epitope of *α*_v_*β*_3_ and *α*_v_*β*_5_ integrin receptors [17]. The radiolabelling of NGR and RGD with ^68^Ga (e.g., ^68^Ga-NOTA-c(NGR) and ^68^Ga-NODAGA-[c(RGD)]_2_) permits the specific imaging of the molecules of neoangiogenesis in the tumors *in vivo* [25]. Previous studies have shown that NGR peptide has high selectivity and specificity for APN/CD13, three times more efficient in the detection of neoangiogenic vessels than RGD [26, 27]; moreover, the cyclic form of NGR is ten times as effective in target detection as the linear form [28, 29].

In this present study, we hypothesized that the expression of APN/CD13 and the development of hypoxia vary during the growth of subcutaneous hepatocellular carcinoma (He/De) in rats. The aim of this study was to evaluate whether ^68^Ga-NOTA-c(NGR) and ^68^Ga-DOTA-nitroimidazole allow the *in vivo* noninvasive detection of the temporal changes of APN/CD13 expression and hypoxia in experimental He/De tumors using positron emission tomography.

## 2. Materials and Methods

### 2.1. Radiopharmacons

^68^Ga-NOTA-c(NGR), ^68^Ga-NODAGA-[c(RGD)]_2_, and ^68^Ga-DOTA-nitroimidazole radiopharmacons (Supplementary data 1: Figure 1) were synthesized according to the description of Máté et al. and Hoigebazar et al. [29, 30] at the University of Debrecen, Department of Medical Imaging. The products were used for PET imaging in sterile form after the quality control.

### 2.2. Cell Cultures

He/De (chemically induced rat hepatocellular carcinoma) cells [31] were cultured in T-75 culture flasks (Sarstedt Ltd., Budapest) with 12 ml of IMDM (Thermo Fisher Scientific Inc., USA) supplemented with 1% antibiotic-antimycotic solution (Thermo Fisher Scientific Inc., USA) and 10% foetal bovine serum (FBS, Thermo Fisher Scientific Inc., USA) at 37°C, in 5% CO_2_ atmosphere and 95% humidity. The cells were used for tumor induction after five passages. The cell viability was determined with a trypan blue exclusion test.

### 2.3. Experimental Animals

16-week-old, 250-300 g weighted male Fischer-344 rats (*n* = 35; Animalab Ltd, Budapest, Hungary) were used for the measurements. The animals were housed under conventional conditions at 23 ± 2°C with 50 ± 10% humidity and artificial lighting with a circadian cycle of 12 h. The semisynthetic diet (VRF1; Akronom Ltd., Budapest, Hungary) and drinking water were available ad libitum to all animals. The animal experiments were authorized by the Ethical Committee for Animal Research, University of Debrecen, Hungary (permission number: 8/2016/DEMÁB). Laboratory animals were kept and treated in compliance with all applicable sections of the Hungarian Laws and animal welfare directions and regulations of the European Union.

### 2.4. Subcutaneous Tumor Transplantation

Fischer-344 rats (*n* = 25) were anaesthetized with a dedicated small animal anaesthesia device (Eickemeyer Tec3 Isoflurane Vaporizer), the left scapular region was depilated and disinfected, and 5 × 10^6^ He/De cells in 150 *μ*l saline were injected subcutaneously.

### 2.5. In Vivo PET Imaging

He/De tumor-bearing animals were anaesthetized with dedicated small animal anaesthesia device (Tec3 Isoflurane Vaporizer) and were injected with 10.2 ± 1.1 MBq of ^68^Ga-NODAGA-[c(RGD)]_2_ or ^68^Ga-NOTA-c(NGR) (angiogenesis imaging) or ^68^Ga-DOTA-nitroimidazole (hypoxia imaging) in 150 *μ*l saline via the lateral tail vein. 90 min after radiotracer injection, whole-body PET/MRI scans were peformed with a preclinical *nanoScan* PET/MRI device (Mediso LTD., Hungary). A special rat imaging chamber (MultiCell Imaging Chamber, Mediso LTD., Hungary) was used to eliminate the movement of rats and to maintain a permanent body temperature. For the anatomical localization of tissues and organs, T1-weighted MRI scans (GRE EXT multi-FOV; phase: 144; TR/TE 15/2 ms; FOV 60 mm; excitation number: 2) were performed. For the image reconstruction (3D-OSEM) and analysis of the PET images, Nucline and InterView™ FUSION software (Mediso LTD., Hungary) were used, respectively. Standardized uptake value (SUV) was calculated through the following formula: SUV = [ROI activity (MBq/ml)]/[injected activity (MBq)/animal weight (g)].

### 2.6. Blocking Experiments

For blocking experiments, He/De tumor-bearing rats were injected with 200 *μ*g unlabelled NOTA-c(NGR) in 100 *μ*l saline via the lateral tail vein five minutes before the injection of ^68^Ga-NOTA-c(NGR). After 90 minutes of incubation time, *in vivo* PET imaging studies were performed with ^68^Ga-labelled tracer, as described above.

### 2.7. Western Blot Analysis

Similar to our previous study [29], for western blot analysis, frozen tissue samples were pulverized under liquid nitrogen and tissue homogenization was performed with TissueLyser II (QIAGEN). Cells were lysed in RIPA buffer (50 mM Tris, 150 mM NaCl, 0.1% SDS, 1% TritonX 100, 0.5% sodium deoxycolate, 1 mM EDTA, 1 mM Na_3_VO_4_, 1 mM NaF, 1 mM PMSF, and protease inhibitor cocktail). After tissue homogenization, the samples were subjected to protein isolation. Protein samples (10-40 *μ*g) were separated on 10% SDS polyacrylamide gels and electrotransferred onto nitrocellulose membranes. After blocking for 1 h with TBST containing 5% BSA, the membranes were incubated with primary antibodies (dilution: 1 : 1000; mouse-anti-rat CD13 and integrin alphaV/beta3 (23C6) (from Santa-Cruz Biotechnology Inc., USA)) overnight at 4°C. After washing with 1x TBST solution, the membranes were probed with IgG HRP-conjugated secondary antibody (Cell Signaling Technology, Inc., Beverly, MA, 1 : 2000). Bands were visualized by enhanced chemiluminescence reaction (SuperSignal West Pico Solutions, Thermo Fisher Scientific Inc., Rockford, USA). Densitometry was performed using ImageJ software. Upon densitometry, negative control samples (rat large intestine) were considered to be 1 and values are expressed as fold change relative to controls to reduce unwanted variations. Beta-actin was used as a loading control.

### 2.8. Statistical Analysis

Experimental data was presented as the mean ± SD of at least three independent experiments. The significance was calculated by Student's *t*-test (two-tailed), two-way ANOVA, and Mann-Whitney *U* test. The significance level was set at *p* ≤ 0.05 unless otherwise indicated.

## 3. Results

### 3.1. *In Vivo* Imaging Studies of He/De Tumors Using ^68^Ga-NOTA-c(NGR)

**Figure 1:**
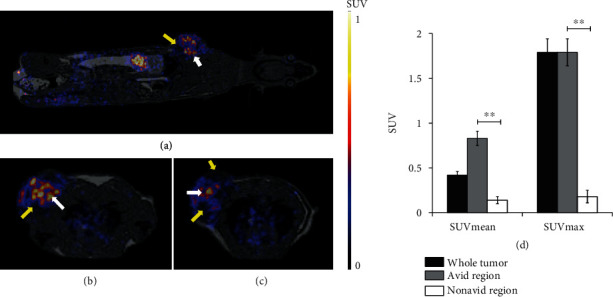
*In vivo* PET/MRI imaging of CD13 expression in subcutaneously transplanted He/De tumors. Representative coronal (a) and axial (b, c) decay-corrected PET/MRI images of subcutaneously transplanted He/De tumors 13 days after tumor induction and 90 min after intravenous injection of ^68^Ga-NOTA-c(NGR). White arrows: CD13 positive, avid regions; yellow arrows: nonavid regions. (d) Quantitative image analysis of heterogenous He/De tumors (*n* = 15). Significance level: *p* ≤ 0.01 (∗∗). Data is presented as the mean ± SD.

In this present study, one of the main objectives was to confirm that ^68^Ga-NOTA-c(NGR) is a suitable radiotracer for imaging of neoangiogenesis in experimental He/De tumors. 90 min after intravenous injection of the CD13-specific ^68^Ga-NOTA-c(NGR), PET/MRI scans were performed. By analyzing the accumulation of ^68^Ga-NOTA-c(NGR) in the whole tumor, we found that the SUVmean and SUVmax values of the He/De tumors were 0.42 ± 0.04 and 1.79 ± 0.15, respectively (Figure 1(d)). Qualitative and quantitative analysis of the decay-corrected PET images showed heterogenous radiotracer accumulation in the subcutaneously growing He/De tumors. By the qualitative analysis of PET images, we found high differences in the accumulation of the radiotracer in the tumors (Figure 1). The quantitative SUV values confirmed this visible heterogeneity. Heterogenous radiotracer uptake (CD13: avid (+) regions and nonavid (-) regions) was observed in He/De tumors, where the SUVmean and SUVmax values were 0.14 ± 0.04 and 0.18 ± 0.07 in the nonavid regions (CD13 negative), furthermore, 0.83 ± 0.08 and 1.79 ± 0.15 in the avid (CD13 positive) regions of He/De tumors. The difference between the SUV data of the avid and nonavid regions was significant at *p* ≤ 0.01 (Figure 1(d)). The APN/CD13 specificity of ^68^Ga-NOTA-c(NGR) was confirmed by blocking experiments using *in vivo* PET imaging and *ex vivo* biodistribution studies (Supplementary data 2: Figure 2 and Supplementary data 3: Table 1).

### 3.2. *In Vivo* Imaging of He/De Tumors Using ^68^Ga-DOTA-Nitroimidazole

**Figure 2:**
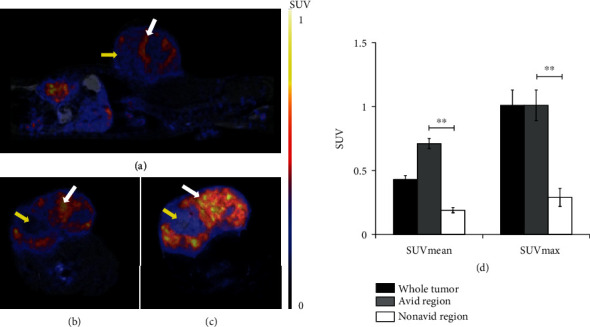
*In vivo* PET/MRI studies of He/De tumors using ^68^Ga-DOTA-nitroimidazole. Representative sagittal (a) and axial (b, c) decay-corrected PET/MRI images of subcutaneous He/De tumors 14 days after tumor transplantation and 90 min after intravenous injection of ^68^Ga-DOTA-nitroimidazole. White arrows: hypoxic regions (avid), yellow arrows: nonhypoxic regions (nonavid). (d) Quantitative SUV analysis of syngeneic He/De tumors (*n* = 15). Significance level: *p* ≤ 0.01. Data is presented as the mean ± SD.

For the detection of hypoxic regions of the subcutaneously transplanted He/De tumors, PET/MRI imaging was performed using hypoxia-specific ^68^Ga-DOTA-nitroimidazole. By the qualitative analysis of PET/MRI images, we found heterogenous radiotracer accumulation in subcutaneously transplanted He/De tumors 14 days after tumor induction (Figure 2). Radiotracer avid (high accumulation) and nonavid regions were sharply distinguishable in the tumors (Figures 2(a)–2(c)). After the quantitative SUV analysis of the PET images, we found that the SUVmean and SUVmax values were 0.43 ± 0.03 and 1.01 ± 0.12, respectively, in the whole tumor (Figure 2(d)). The heterogeneity of experimental tumors was also confirmed by the quantitative analysis of ^68^Ga-DOTA-nitroimidazole accumulation in avid and nonavid regions. SUVmean and SUVmax values were 0.71 ± 0.04 and 1.01 ± 0.12, respectively, in the avid regions of He/De tumors. Furthermore, the SUV values were significantly (*p* ≤ 0.01) lower in the nonavid regions where the SUVmean and SUVmax values were 0.19 ± 0.02 and 0.29 ± 0.07, respectively (Figure 2(d)).

### 3.3. *In Vivo* Imaging Studies of He/De Tumors Using ^68^Ga-NODAGA-[c(RGD)]_2_

**Figure 3:**
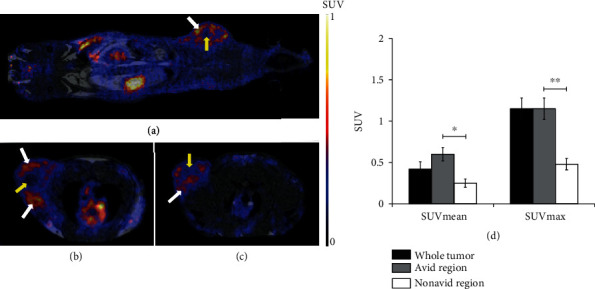
*In vivo* PET/MRI studies of *α*_v_*β*_3_ integrin receptor expression in subcutaneously transplanted He/De tumors. Representative coronal (a) and axial (b, c) PET/MRI images of subcutaneously transplanted He/De tumors 13 days after tumor induction and 90 min after intravenous injection of ^68^Ga-NODAGA-[c(RGD)]_2_. White arrows: *α*_v_*β*_3_ integrin receptor positive (avid) regions; yellow arrows: nonavid regions. (d) Quantitative PET/MRI image analysis of heterogenous He/De (*n* = 15) tumors. Significance levels: *p* ≤ 0.05 (∗) and *p* ≤ 0.01 (∗∗). Data is presented as the mean ± SD.

For the *in vivo* determination of *α*_v_*β*_3_ integrin expression of subcutaneously transplanted He/De tumors, PET/MRI scans were performed 90 min after intravenous injection of ^68^Ga-NODAGA-[c(RGD)]_2_. By the qualitative analysis of the decay-corrected PET images, He/De tumors were clearly identifiable with the SUVmean and SUVmax values of 0.42 ± 0.09 and 1.15 ± 0.13, respectively (Figure 3). Moreover, heterogeneity in ^68^Ga-NODAGA-[c(RGD)]_2_ accumulation was also demonstrated in the He/De tumors. After the quantitative SUV analysis of the radiotracer avid regions of subcutaneously growing He/De tumors, we found that the SUVmean and SUVmax values were 0.60 ± 0.08 and 1.15 ± 0.13, respectively. These SUV values were approximately twofold higher than that of the nonradiotracer avid regions were the SUVmean was 0.25 ± 0.05 and the SUVmax was 0.48 ± 0.07. The difference between the avid and nonavid regions of the tumors was significant (*p* ≤ 0.01) (Figure 3(d)).

### 3.4. *In Vivo* Imaging Study of Hypoxia and Angiogenesis in correspondence with Tumor Volume Enlargement in Subcutaneously Transplanted He/De Tumors

**Figure 4:**
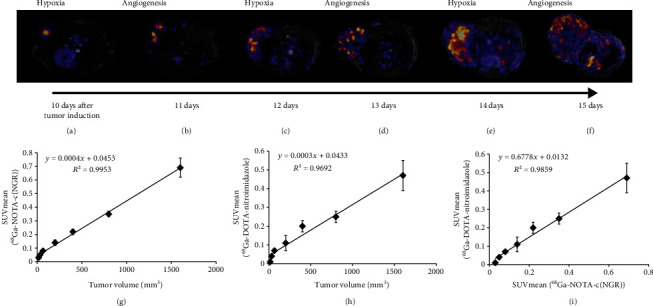
*In vivo* PET/MRI image analysis of hypoxia regions and CD13 expression in relation to tumor volume enlargement in subcutaneously transplanted He/De tumors. Representative axial PET/MRI images of subcutaneously transplanted He/De tumor-bearing rats 90 min after intravenous injection of ^68^Ga-DOTA-nitroimidazole (a, c, e) and ^68^Ga-NOTA-c(NGR) (b, d, f). Quantitative analysis of PET/MRI images of He/De tumors using ^68^Ga-NOTA-c(NGR) (g) and ^68^Ga-DOTA-nitroimidazole (h) in correlation with tumor volume enlargement and relation between ^68^Ga-NOTA-c(NGR) and ^68^Ga-DOTA-nitroimidazole during tumor growth (i). Data is presented as the mean ± SD.

For the *in vivo* investigation of the relationship between neoangiogenesis and hypoxia in correspondence with tumor volume enlargement in He/De tumors, PET/MRI scans were performed 90 min after the intravenous injection of radiotracers. Subcutaneously transplanted He/De tumors were inspected day by day by turns using ^68^Ga-DOTA-nitroimidazole for imaging hypoxia and ^68^Ga-NOTA-c(NGR) for imaging angiogenesis (Figure 4). Qualitative PET/MRI image analysis showed an increased level of hypoxia and neoangiogenesis related to tumor volume enlargement. Changes in the size of hypoxic and neoangiogenic regions were correlated with the tumor volume which was confirmed by the quantitative SUV data. The uptake of ^68^Ga-NOTA-c(NGR) and ^68^Ga-DOTA-nitroimidazole increased with the enlargement of tumor volume. SUVmean values of the whole tumor mass were used for the quantitative analysis. At approximately 16, 33, 66, 200, 400, 800, and 1600 mm^3^ of tumor volume, the SUVmean values of ^68^Ga-NOTA-c(NGR) were 0.03 ± 0.01, 0.05 ± 0.01, 0.08 ± 0.004, 0.14 ± 0.02, 0.22 ± 0.02, 0.35 ± 0.02, and 0.69 ± 0.07, respectively. ^68^Ga-NOTA-c(NGR) uptake correlated (*R*^2^ = 0.9953) with tumor growth (Figure 4(g)). Similar results were observed when ^68^Ga-DOTA-nitroimidazole was used where the SUVmean values were 0.01 ± 0.004, 0.04 ± 0.005, 0.07 ± 0.01, 0.11 ± 0.04, 0.20 ± 0.03, 0.25 ± 0.03, and 0.47 ± 0.08 at approx. 16, 33, 66, 200, 400, 800, and 1600 mm^3^ of tumor volume, respectively. The increasing of tumor volume also correlated well (*R*^2^ = 0.9692) with the elevated uptake of ^68^Ga-DOTA-nitroimidazole (Figure 4(h)). By analyzing the accumulation of the two radiopharmaceuticals during tumor growth, we found that they show an increasing uptake in close correlation with each other (Figure 4(i)). Data obtained from *in vivo* PET observations was confirmed by *ex vivo* measurements (Supplementary data 3: Table 1).

### 3.5. Western Blot Analysis

**Figure 5:**
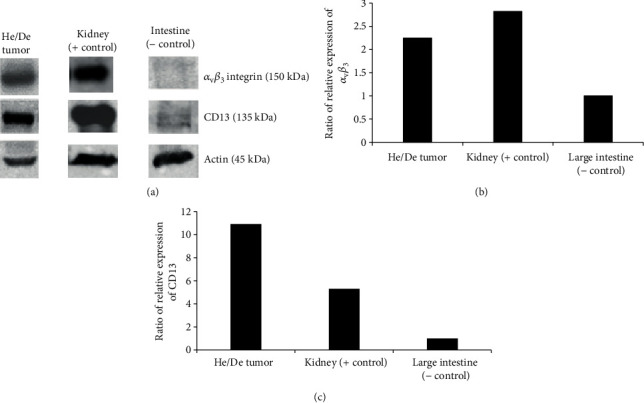
Qualitative (a) and quantitative western blot analysis of *α*_v_*β*_3_ integrin (b) and CD13 (c) expression in subcutaneously transplanted He/De tumors. In densitometry, negative control samples (rat large intestine) were considered to be 1, and values are expressed as fold change relative to controls.

The expression of *α*_v_*β*_3_ integrin and CD13 was verified by western blot analysis in subcutaneously transplanted He/De tumors (Figure 5). We found that among the investigated neoangiogenic markers, the expression of APN/CD13 showed strong positivity.

## 4. Discussion

Confirming the presence of hypoxia and of neoangiogenic markers in tumors by *in vivo* PET imaging using specific radiopharmaceuticals can greatly assist in the selection of appropriate antitumor therapy. *In vivo* imaging of tumor hypoxia and neoangiogenesis is an intensively researched area in the field of nuclear medicine and radiotracer development. Hypoxia influences tumor resistance to radio- or chemotherapy; moreover, hypoxia is a major stimulator of expression of different neoangiogenic molecules, *e.g.*, VEGF and CD13, in tumors [32]. Neovascularization of malignant tumors plays an important role in tumor growth, tumor progression, and the efficacy of antitumor therapies based on antiangiogenic molecules [33–35].

In the human clinical routine tumor imaging, *e.g.*, ^18^F-FDG, ^18^F-FLT, or ^11^C-methionine [36, 37], radiotracers are widely used for the detection of tumors and metastases; however, these radiotracers are not specific for hypoxia or angiogenic molecules. Furthermore, it is known that ^18^F-FDG has low accumulation and poor diagnostic efficiency in well-differentiated hepatocellular carcinoma. Based on this property, it is less suitable to demonstrate the efficacy of an antitumor therapy [38]. Due to this, in our study, ^68^Ga-DOTA-nitroimidazole was used for specific imaging of tumor hypoxia and ^68^Ga-NODAGA-[c(RGD)]_2_ and ^68^Ga-NOTA-c(NGR) for imaging of neoangiogenesis in experimental hepatocellular carcinoma tumors in correspondence with tumor volume enlargement.

The first step in our study was the *in vivo* identification of hypoxia regions in He/De tumors with PET/MRI imaging. It is known that partial pressure of oxygen (pO_2_) is reduced in hypoxic regions of tumors which results in the accumulation of the nitroimidazole molecule and its derivatives in hypoxic tumor cells. In our experiments, 10 days after tumor cell inoculation, high ^68^Ga-DOTA-nitroimidazole uptake was already observed in subcutaneously transplanted He/De tumors at the tumor size of 125 mm^3^. Furthermore, from 11 days after the implantation of He/De cells, hypoxic (^68^Ga-DOTA-nitroimidazole avid) regions were clearly discerned in the tumor mass with significantly higher (*p* ≤ 0.01) SUV values than that of the normoxic or necrotic regions (Figure 2). This heterogeneity in the tumor mass was also described by other research groups, and they found that its rate can be 50-60% of the whole tumor [39, 40]. Henceforward, in our *in vivo* experiments, the expression of APN/CD13 and *α*_v_*β*_3_ integrin receptors as angiogenesis markers was investigated with PET/MRI in the He/De tumor model. It is known from other papers that these two molecules are functioning as receptors and are overexpressed on the surface of endothelial cells. Previous studies have shown that NGR peptides specifically bind to APN/CD13, and RGD molecules are the ligands of *α*_v_*β*_3_ integrin receptors; in addition, the ^68^Ga-labelled NGR and RGD peptides are useful radiotracers for the *in vivo* PET imaging of neoangiogenic processes in tumors [25, 27, 29, 41]. However, our research group previously reported that the uptake of ^68^Ga-NOTAc(NGR) of the primary tumors was significantly higher than that of the accumulation of the commercially available ^68^Ga-NODAGA-[c(RGD)]_2_ in the same tumor, when experimental renal (Ne/De) tumors were investigated [29]. In our hepatocellular carcinoma model, this difference between the uptake of ^68^Ga-NODAGA-[c(RGD)]_2_ and ^68^Ga-NOTA-c(NGR) in He/De tumors was also found. This observation is due to the higher expression of APN/CD13 in He/De tumors which was confirmed by western blot analysis (Figure 5). However, in this present study, *in vivo* PET images of He/De tumors showed strong heterogeneity in the accumulation of ^68^Ga-NODAGA-[c(RGD)]_2_ and ^68^Ga-NOTA-c(NGR) in subcutaneously transplanted He/De tumors. Similar to the results of our hypoxia imaging studies, significantly higher (*p* ≤ 0.01) SUV values were observed in the APN/CD13 and *α*_v_*β*_3_ integrin positive regions of the He/De tumors than those of the negative areas using both angiogenesis-specific radiotracers. This observation of tumor heterogeneity is known since the angiogenic phenotype can be extraordinarily diverse within the same tumor due to hypoxia or tumor-associated inflammatory processes (TNF*α*, TGF*β*, and IL-6) taking effect on angiogenesis. All these effects with genetic instability result in abnormal, heterogenous angiogenesis [42].

Tumor enlargement is greatly influenced by the oxygen and nutrient supply. In case of reduced oxygen supply (hypoxia), HIF transcriptional factors are activated which promotes gene expression of those responsible for tumor survival and progression. One of these hypoxia and HIF-induced processes is tumor neoangiogenesis [43]. In this present study, for the *in vivo* assessment of the correspondence between angiogenesis and tumor growth, ^68^Ga-NOTA-c(NGR) was used due to the fact that the expression of APN/CD13 was higher than that of *α*_v_*β*_3_ integrin in He/De tumors. We hypothesized that the hypoxic and angiogenic areas increase in size with tumor volume. This assumption was confirmed since ^68^Ga-NOTA-c(NGR) and ^68^Ga-DOTA-nitroimidazole uptake increased and showed a strong correlation with the tumor volume enlargement (Figure 4 and Supplementary data 3: Table 1). Furthermore, we hypothesized that elevation of hypoxia will induce increasingly strong angiogenesis with tumor growth. This was confirmed by indirect evidence that the increasing uptake of ^68^Ga-DOTA-nitroimidazole was strongly correlated with the accumulation of ^68^Ga-NOTA-c(NGR) (Figure 4). Interestingly, Deshpande et al. [44] found the opposite when integrin, endoglin, and VEGFR2 expression levels of different tumors were followed with targeted microbubbles by ultrasound imaging in tumors of different sizes. They found that the expression of these angiogenic markers decreased with increasing size of tumor xenografts. An explanation for this phenomenon may be the matrigel used during the injection of tumor cells, which is known to have an angiogenesis-inducing effect. Furthermore, they described that the site of tumor cell transplantation and the applied preclinical model also influence the time appearance of angiogenic markers; therefore, it is necessary to follow the change in the expression of angiogenic markers over time in as many tumor models as possible.

The correlation between hypoxia and angiogenesis was already investigated by other workgroups. In general view, angiogenesis is the outcome of hypoxia because the oxygen applied is increased, and it results in new blood vessel formation [2, 6, 45, 46]. However, by investigation of other diseases (*e.g.*, liver fibrosis and wound repair), it was pointed out that the relationship between hypoxia and angiogenesis is more complicated than it was earlier imagined. Hypoxia can also be the cause or the consequence of tissue damage. Hypoxia is not necessarily the result of blood supply, but rather, it can also be the result of the proliferation and inflammatory response; furthermore, hypoxia is the result of the contradiction between oxygen supply and need [47–49].

In tumors, hypoxia can be developed without any connection to oxygen supply. Oxygen utilization increases fivefold during cell proliferation; consequently, hypoxia-induced angiogenesis also occurs at small tumor size. Small tumors consist of approximately 100 cells can induce angiogenesis by the reduced pO_2_ level due to the increased oxygen consumption. In this present work, we also found that hypoxic regions can be observed in He/De tumors at a small size by ^68^Ga-DOTA-nitroimidazole imaging (Figure 4(a)).

In summary, as our research group previously demonstrated for Ne/De renal tumors [29], ^68^Ga-NOTA-c(NGR) is a suitable diagnostic agent for the detection of APN/CD13 expression in a He/De hepatocellular carcinoma tumor also. APN/CD13 and hypoxia-specific radiopharmaceuticals may contribute significantly to a better understanding of the relationship between hypoxia and neoangiogenesis in tumors. Therefore, the *in vivo* detection of the presence of angiogenic molecules—as potential therapeutic targets—and the determination of the changes in their expression levels during tumor growth may play an important role in the development of antitumor therapies.

## 5. Conclusion

Noninvasive *in vivo* PET/MRI imaging using ^68^Ga-NOTA-c(NGR) and ^68^Ga-DOTA-nitroimidazole—as specific radiolabelled diagnostic molecules—provides an opportunity to the detection of temporal changes of hypoxic regions and neoangiogenic molecule (APN/CD13) expression in hepatocellular carcinoma tumors. These results give the possibility of the development of new molecular imaging strategies, the early detection of tumors, and the monitoring of antitumor therapies.

## Data Availability

The data used to support the findings of this study are available from the corresponding author upon request.

## Abbreviations

APN/CD13: Aminopeptidase N
He/De: Hepatocellular carcinoma
NGR: Asparaginyl-glycyl-arginine
PET: Positron emission tomography
RGD: Argininyl-glycinyl-aspartic acid
SUV: Standardized uptake value
T/M: Tumor-to-muscle ratio.

## Supplementary Materials

Supplementary Materials Supplementary data 1: Figure 1: chemical structures of ^68^Ga-NODAGA-[c(RGD)]_2_ (A), ^68^Ga-NOTA-c(NGR) (B), and ^68^Ga-DOTA-nitroimidazole (C). Supplementary data 2: Figure 2: representative decay-corrected axial PET/MRI images of subcutaneously transplanted He/De tumors (red arrows) 90 min after intravenous injection of ^68^Ga-NOTA-c(NGR). A: basic, B: blocked with unlabelled NOTA-c(NGR), and C and D: quantitative SUV analysis of ^68^Ga-NOTA-c(NGR) accumulation in He/De tumors (*n* = 10/group). T/M: tumor-to-muscle ratio. Significance level: *p* ≤ 0.01 (∗∗). Data is presented as the mean ± SD. Supplementary data 3: Table 1: ex vivo biodistribution (%ID/g) of ^68^Ga-NOTA-c(NGR), ^68^Ga-DOTA-nitroimidazole, and ^68^Ga-NODAGA-[c(RGD)]_2_ in He/De tumors 90 min after tracer injection and 9 ± 1, 12 ± 1, and 15 ± 1 days after subcutaneous tumor induction.
