# Supplementary material for: In Vivo Imaging of Hypoxia and Neoangiogenesis in Experimental Syngeneic Hepatocellular Carcinoma Tumor Model Using Positron Emission Tomography
Source: Biomed Res Int. 2020 Aug 7;2020:4952372. doi: 10.1155/2020/4952372 (PMC7428931; doi:10.1155/2020/4952372)
Supplement: Supplementary Materials — Supplementary data 1: Figure 1: chemical structures of 68Ga-NODAGA-[c(RGD)]2 (A), 68Ga-NOTA-c(NGR) (B), and 68Ga-DOTA-nitroimidazole (C). Supplementary data 2: Figure 2: representative decay-corrected axial PET/MRI images of subcutaneously transplanted He/De tumors (red arrows) 90 min after intravenous injection of 68Ga-NOTA-c(NGR). A: basic, B: blocked with unlabelled NOTA-c(NGR), and C and D: quantitative SUV analysis of 68Ga-NOTA-c(NGR) accumulation in He/De tumors (n = 10/group). T/M: tumor-to-muscle ratio. Significance level: p ≤ 0.01 (∗∗). Data is presented as the mean ± SD. Supplementary data 3: Table 1: ex vivo biodistribution (%ID/g) of 68Ga-NOTA-c(NGR), 68Ga-DOTA-nitroimidazole, and 68Ga-NODAGA-[c(RGD)]2 in He/De tumors 90 min after tracer injection and 9 ± 1, 12 ± 1, and 15 ± 1 days after subcutaneous tumor induction. [file 4952372.f1.zip › 4952372.f1/Supplementary Material Kis_et_al_2020_4952372 FINAL.docx]

**Supplementary Material**

**Supplementary data 1:**

**1. Radiopharmaceuticals**


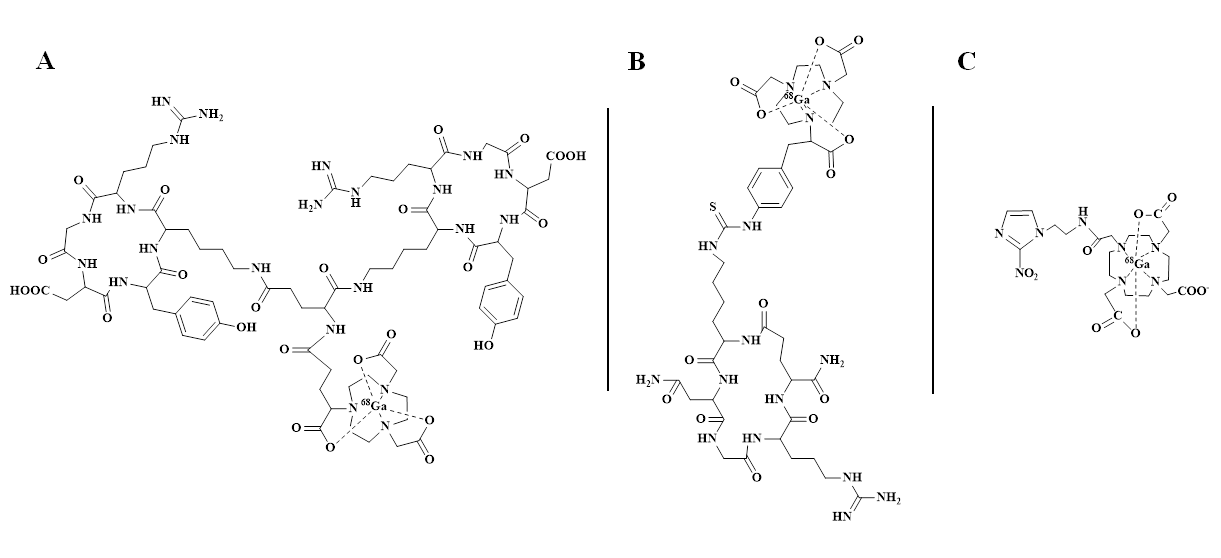


**Figure. 1** Chemical structures of ^68^Ga-NODAGA-[c(RGD)]_2_ (A), ^68^Ga-NOTA-c(NGR) (B) and ^68^Ga-DOTA-Nitroimidazole (C).

**Supplementary data 2:**

**2. *In vivo* blocking experiments**

The APN/CD13 specificity of ^68^Ga-NOTA-c(NGR) was confirmed by blocking experiments using *in vivo* PET imaging studies (Supplementary Material, Fig. 2). He/De tumor bearing rats were injected with 200 µg unlabelled NOTA-c(NGR) (approx. 100-fold of the radiolabelled peptide) prior to ^68^Ga-NOTA-c(NGR) injection. The accumulation of ^68^Ga-NOTA-c(NGR) in He/De tumors decreased after the administration of unlabelled NOTA-c(NGR) (Fig. 2B). Quantitative SUV data analysis showed that significantly (p≤0.01) lower SUVmean (0.04±0.01), SUVmax (0.05±0.01), T/M SUVmean (2.14±0.89) and T/M SUVmax (3.45±1.11) values were observed using the unlabelled NOTA-c(NGR) than that of the absence of the cold material, where the T/M SUVmean and T/M SUVmax values of He/De tumors were 11.23±1.15 and 12.62±1.44, respectively (Fig. 2C and D). These results verified the APN/CD13 specificity of ^68^Ga-NOTA-c(NGR).


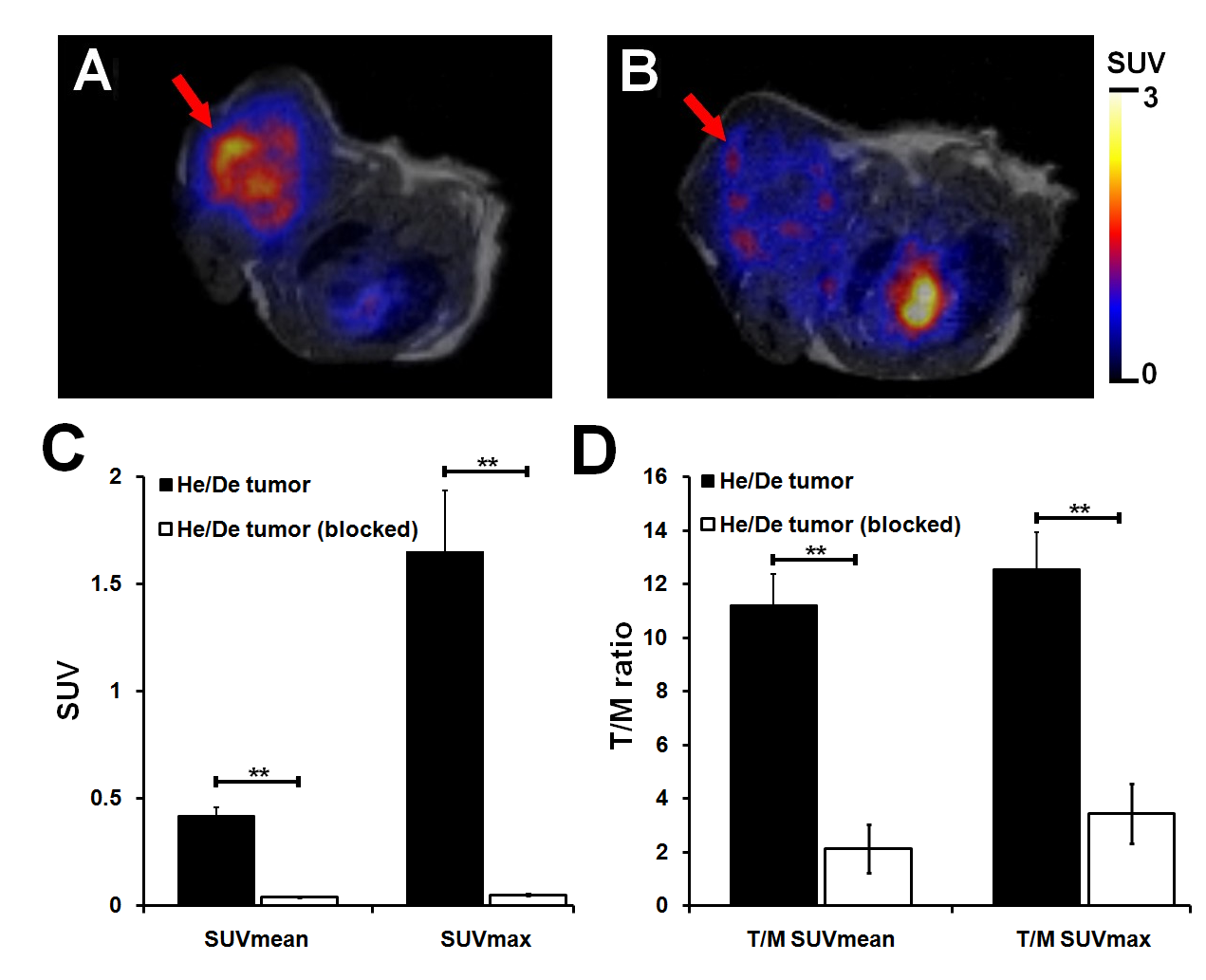


Figure 2. Representative decay-corrected axial PET/MRI images of subcutaneously transplanted He/De tumors (red arrows) 90 min after intravenous injection of ^68^Ga-NOTA-c(NGR). A: basic, B: blocked with unlabelled NOTA-c(NGR); C and D: quantitative SUV analysis of ^68^Ga-NOTA-c(NGR) accumulation in He/De tumors (n=10/group). T/M: tumor-to-muscle ratio. Significance level: p≤0.01 (**). Data is presented as mean±SD.

**Supplementary data 3:**

**3. *Ex vivo* biodistribution studies**

For *ex vivo* biodistribution studies approx. 10 MBq of ^68^Ga-NOTA-c(NGR), ^68^Ga-DOTA-Nitroimidazole or ^68^Ga-NODAGA-[c(RGD)]_2_ was injected via the lateral tail vein into He/De tumor-bearing rats and, after 90 min incubation time rats were euthanized with 5% Forane. The weight and the radioactivities of the whole tumors and tissue samples (muscle as background) were measured with calibrated gamma counter and the uptake was expressed as %ID/g tissue. Table 1 shows an increasing radiotracer uptake in dynamically growing He/De tumors using all of the three radiotracers. In blocking experiments, the %ID values of ^68^Ga-NOTA-c(NGR) significantly (*p*≤0.01) decreased in He/De tumors by using unlabelled NOTA-c(NGR). This observation signed that the tracer uptake of the tumor was blocked efficiently, confirming the CD13 binding specificity of ^68^Ga-NOTA-c(NGR).

**Table 1**

*Ex vivo* biodistribution (%ID/g) of ^68^Ga-NOTA-c(NGR), ^68^Ga-DOTA-Nitroimidazole and ^68^Ga-NODAGA-[c(RGD)]_2_ in He/De tumors 90 min after tracer injection and 9±1, 12±1, and 15±1 days after subcutaneous tumor induction. Significance level between blocked and non-blocked He/De tumors at 90 min: *p*≤0.01 (**). 200 μg unlabelled NOTA-c(NGR) was used for blocking. T/M: tumor-to-muscle ratio.

| **Tumor** | **^68^Ga-NOTA-c(NGR)**  (n=3) | **^68^Ga-DOTA-Nitroimidazole**  (n=3) | **^68^Ga-NODAGA-[c(RGD)]_2_**  (n=3) |
| --- | --- | --- | --- |
| He/De (small tumor, 9±1 days) | 0.10 ± 0.01 | 0.12 ± 0.05 | 0.09 ± 0.01 |
| He/De (medium tumor, 12±1 days) | 0.23 ± 0.09 | 0.21 ± 0.06 | 0.16 ± 0.03 |
| He/De (large tumor, 15±1 days) | 0.41 ± 0.08** | 0.28 ± 0.04 | 0.31 ± 0.05 |
| He/De blocked (15±1 days) | 0.09 ± 0.03 | - | - |
| T/M ratio (15±1 days) | 9.15 ± 1.02 | 5.69 ± 0.40 | 5.35 ± 0.89 |
