# Supplementary material for: In Vivo Imaging of Hypoxia and Neoangiogenesis in Experimental Syngeneic Hepatocellular Carcinoma Tumor Model Using Positron Emission Tomography
Source: Biomed Res Int. 2020 Aug 7;2020:4952372. doi: 10.1155/2020/4952372 (PMC7428931; doi:10.1155/2020/4952372)
Supplement: Supplementary Materials — Supplementary data 1: Figure 1: chemical structures of 68Ga-NODAGA-[c(RGD)]2 (A), 68Ga-NOTA-c(NGR) (B), and 68Ga-DOTA-nitroimidazole (C). Supplementary data 2: Figure 2: representative decay-corrected axial PET/MRI images of subcutaneously transplanted He/De tumors (red arrows) 90 min after intravenous injection of 68Ga-NOTA-c(NGR). A: basic, B: blocked with unlabelled NOTA-c(NGR), and C and D: quantitative SUV analysis of 68Ga-NOTA-c(NGR) accumulation in He/De tumors (n = 10/group). T/M: tumor-to-muscle ratio. Significance level: p ≤ 0.01 (∗∗). Data is presented as the mean ± SD. Supplementary data 3: Table 1: ex vivo biodistribution (%ID/g) of 68Ga-NOTA-c(NGR), 68Ga-DOTA-nitroimidazole, and 68Ga-NODAGA-[c(RGD)]2 in He/De tumors 90 min after tracer injection and 9 ± 1, 12 ± 1, and 15 ± 1 days after subcutaneous tumor induction. [file 4952372.f1.zip › 4952372.f1/Supplementary Material Kis_et_al_2020_4952372 FINAL.pdf]

Supplementary Material

Supplementary data 1:

1. Radiopharmaceuticals

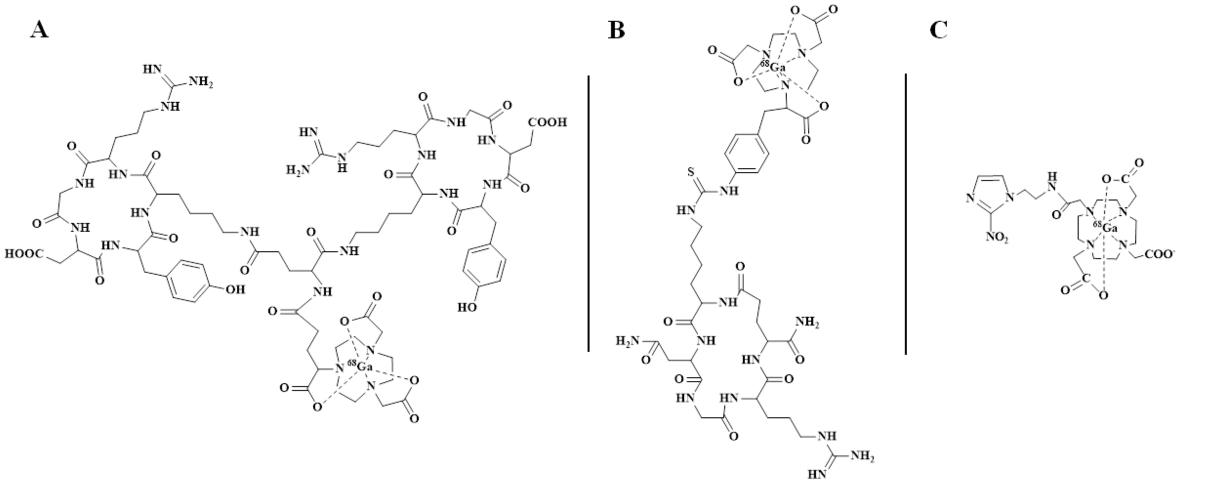

Figure. 1 Chemical structures of ^{68}Ga-NODAGA-[c(RGD)]$_2$ (A), ^{68}Ga-NOTA-c(NGR) (B) and ^{68}Ga-DOTA-Nitroimidazole (C).

Supplementary data 2:

2. *In vivo* blocking experiments

The APN/CD13 specificity of ^{68}Ga-NOTA-c(NGR) was confirmed by blocking experiments using *in vivo* PET imaging studies (Supplementary Material, Fig. 2). He/De tumor bearing rats were injected with 200 µg unlabelled NOTA-c(NGR) (approx. 100-fold of the radiolabelled peptide) prior to ^{68}Ga-NOTA-c(NGR) injection. The accumulation of ^{68}Ga-NOTA-c(NGR) in He/De tumors decreased after the administration of unlabelled NOTA-c(NGR) (Fig. 2B). Quantitative SUV data analysis showed that significantly ($p \leq 0.01$) lower SUVmean (0.04±0.01), SUVmax (0.05±0.01), T/M SUVmean (2.14±0.89) and T/M SUVmax (3.45±1.11) values were observed using the unlabelled NOTA-c(NGR) than that of the absence of the cold material, where the T/M SUVmean and T/M SUVmax values of He/De tumors were 11.23±1.15

and 12.62±1.44, respectively (Fig. 2C and D). These results verified the APN/CD13 specificity of ^{68}Ga-NOTA-c(NGR).

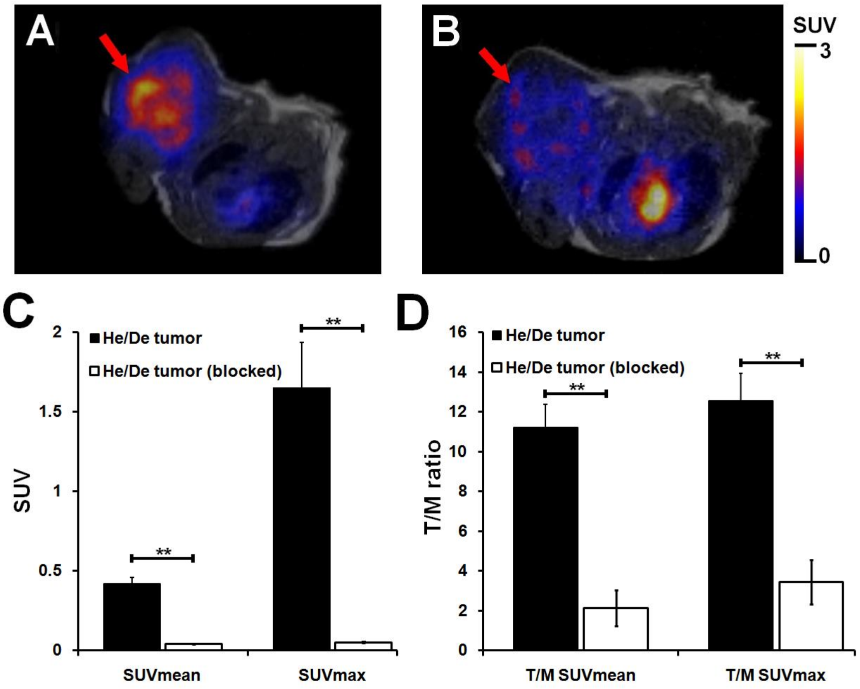

Figure 2. Representative decay-corrected axial PET/MRI images of subcutaneously transplanted He/De tumors (red arrows) 90 min after intravenous injection of ^{68}Ga-NOTA-c(NGR). A: basic, B: blocked with unlabelled NOTA-c(NGR); C and D: quantitative SUV analysis of ^{68}Ga-NOTA-c(NGR) accumulation in He/De tumors (n=10/group). T/M: tumor-to-muscle ratio. Significance level: $p \leq 0.01$ (**). Data is presented as mean±SD.

Supplementary data 3:

3. *Ex vivo* biodistribution studies

For *ex vivo* biodistribution studies approx. 10 MBq of ^{68}Ga-NOTA-c(NGR), ^{68}Ga-DOTA-Nitroimidazole or ^{68}Ga-NODAGA-[c(RGD)]$_2$ was injected via the lateral tail vein into He/De tumor-bearing rats and, after 90 min incubation time rats were euthanized with 5% Forane. The weight and the radioactivities of the whole tumors and tissue samples (muscle as background) were measured with calibrated gamma counter and the uptake was expressed as %ID/g tissue. Table 1 shows an increasing radiotracer uptake in dynamically growing He/De tumors using all

of the three radiotracers. In blocking experiments, the %ID values of ^{68}Ga-NOTA-c(NGR) significantly (p≤0.01) decreased in He/De tumors by using unlabelled NOTA-c(NGR). This observation signed that the tracer uptake of the tumor was blocked efficiently, confirming the CD13 binding specificity of ^{68}Ga-NOTA-c(NGR).

Table 1

Ex vivo biodistribution (%ID/g) of ^{68}Ga-NOTA-c(NGR), ^{68}Ga-DOTA-Nitroimidazole and ^{68}Ga-NODAGA-[c(RGD)]$_2$ in He/De tumors 90 min after tracer injection and 9±1, 12±1, and 15±1 days after subcutaneous tumor induction. Significance level between blocked and non-blocked He/De tumors at 90 min: p≤0.01 (**). 200 µg unlabelled NOTA-c(NGR) was used for blocking. T/M: tumor-to-muscle ratio.

**Tumor**	**^{68}Ga-NOTA-c(NGR)** (n=3)	**^{68}Ga-DOTA-Nitroimidazole** (n=3)	**^{68}Ga-NODAGA-[c(RGD)]$_2$** (n=3)
He/De (small tumor, 9±1 days)	0.10 ± 0.01	0.12 ± 0.05	0.09 ± 0.01
He/De (medium tumor, 12±1 days)	0.23 ± 0.09	0.21 ± 0.06	0.16 ± 0.03
He/De (large tumor, 15±1 days)	0.41 ± 0.08**	0.28 ± 0.04	0.31 ± 0.05
He/De blocked (15±1 days)	0.09 ± 0.03	-	-
T/M ratio (15±1 days)	9.15 ± 1.02	5.69 ± 0.40	5.35 ± 0.89
