# Supplementary figures and images for: In Vivo Imaging of Hypoxia and Neoangiogenesis in Experimental Syngeneic Hepatocellular Carcinoma Tumor Model Using Positron Emission Tomography
Source: Biomed Res Int. 2020 Aug 7;2020:4952372. doi: 10.1155/2020/4952372 (PMC7428931; doi:10.1155/2020/4952372)

## Slide 1
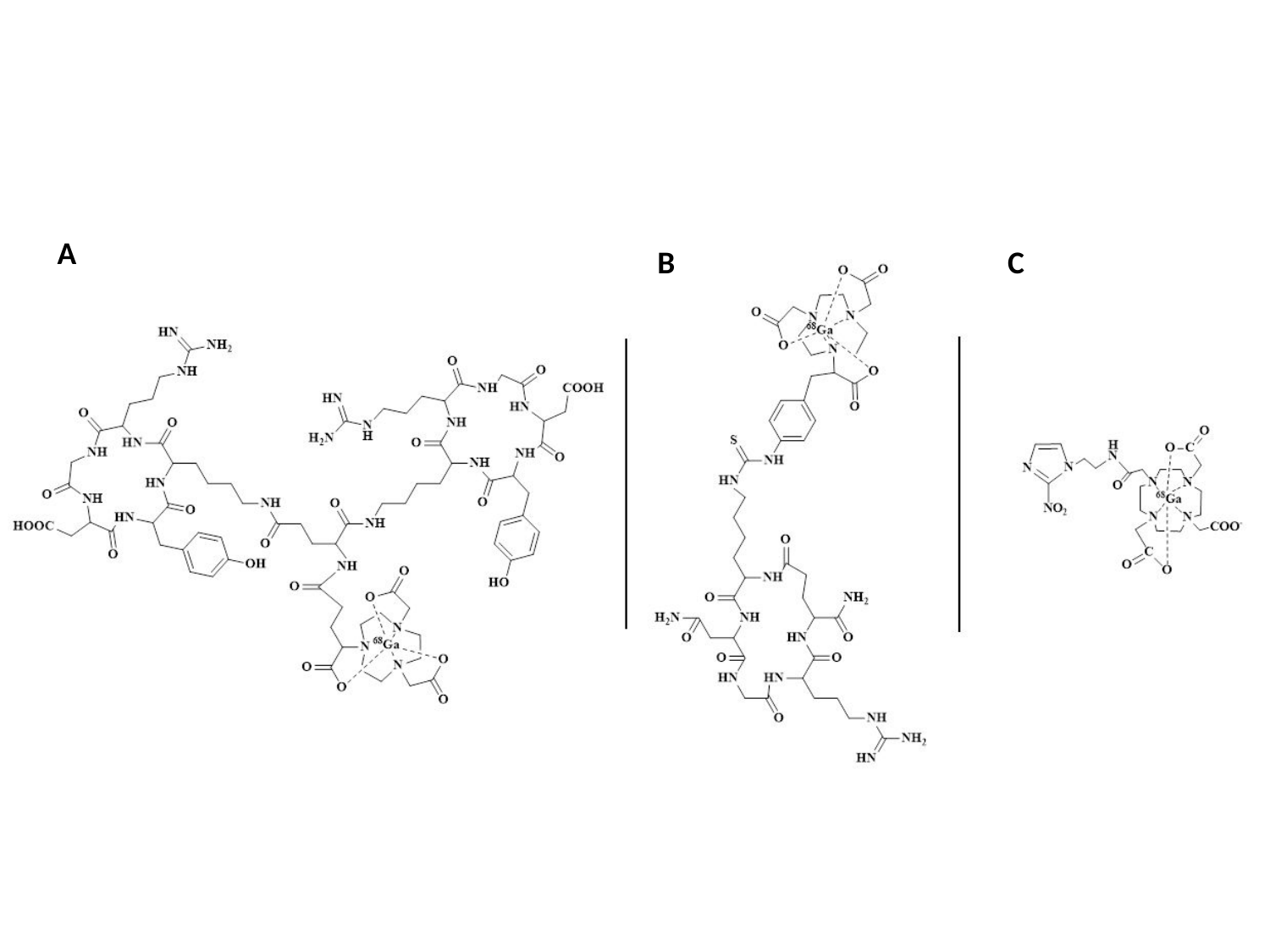

A
B
C

Supplement: Supplementary Materials — Supplementary data 1: Figure 1: chemical structures of 68Ga-NODAGA-[c(RGD)]2 (A), 68Ga-NOTA-c(NGR) (B), and 68Ga-DOTA-nitroimidazole (C). Supplementary data 2: Figure 2: representative decay-corrected axial PET/MRI images of subcutaneously transplanted He/De tumors (red arrows) 90 min after intravenous injection of 68Ga-NOTA-c(NGR). A: basic, B: blocked with unlabelled NOTA-c(NGR), and C and D: quantitative SUV analysis of 68Ga-NOTA-c(NGR) accumulation in He/De tumors (n = 10/group). T/M: tumor-to-muscle ratio. Significance level: p ≤ 0.01 (∗∗). Data is presented as the mean ± SD. Supplementary data 3: Table 1: ex vivo biodistribution (%ID/g) of 68Ga-NOTA-c(NGR), 68Ga-DOTA-nitroimidazole, and 68Ga-NODAGA-[c(RGD)]2 in He/De tumors 90 min after tracer injection and 9 ± 1, 12 ± 1, and 15 ± 1 days after subcutaneous tumor induction. [file 4952372.f1.zip › 4952372.f1/Supplementary Figure 1_Kis_et_al_2020_BMRI_3125570.pptx]
